# Pediatric Drug Poisoning in Vojvodina, Serbia: A Retrospective Observational Clinical and Toxicological Assessment

**DOI:** 10.3390/jcm14175967

**Published:** 2025-08-23

**Authors:** Jovan Baljak, Aleksandra Stojadinović, Dragan Zečević, Maja Đurendić-Brenesel, Nikša Ajduković, Dušan Vapa, Miljana Poparić, David Strilić, Nataša Tomić, Aleksandar Rašković

**Affiliations:** 1 Center for Forensic Medicine, Toxicology and Molecular Genetics, Clinical Center of Vojvodina, Hajduk Veljkova 3, 21000 Novi Sad, Serbia; jovan.baljak@mf.uns.ac.rs (J.B.); maja.djurendic@gmail.com (M.Đ.-B.); niksa_ajdukovic@yahoo.com (N.A.); dusan.vapa@mf.uns.ac.rs (D.V.); 2 Faculty of Medicine, University of Novi Sad, Hajduk Veljkova 3, 21000 Novi Sad, Serbia; miljana.poparic@mf.uns.ac.rs (M.P.); 902027d24@mf.uns.ac.rs (D.S.); 3 Institute for Child and Youth Health Care Vojvodina, Hajduk Veljkova 3, 21000 Novi Sad, Serbia; 4 Department of Pediatrics, Faculty of Medicine, University of Novi Sad, Hajduk Veljkova 3, 21000 Novi Sad, Serbia; 5 International Center for Cardiovascular Diseases, MC Medicor, Polje 40, 6310 Izola, Slovenia; mcmedicor@siol.net; 6 Department of Forensic Medicine, Faculty of Medicine, University of Novi Sad, Hajduk Veljkova 3, 21000 Novi Sad, Serbia; 7 Clinical for Anesthesia, Intensive Care and Pain Therapy, Clinical Center of Vojvodina, Hajduk Veljkova 3, 21000 Novi Sad, Serbia; natasa.tomic@kcv.rs; 8 Department of Pharmacology and Toxicology, Faculty of Medicine, University of Novi Sad, Hajduk Veljkova 3, 21000 Novi Sad, Serbia; aleksandar.raskovic@mf.uns.ac.rs

**Keywords:** drug poisoning, pediatric population, poisoning, benzodiazepines, clinical manifestation

## Abstract

**Objectives:** Acute drug poisoning represents a significant public health issue among the pediatric population. The aim of this study was to evaluate the characteristics of drug poisoning in children and adolescents in the Vojvodina region from 2018 to 2023. **Methods:** In a retrospective observational study, 82 patients with confirmed drug poisoning were included, and data was collected regarding demographic characteristics, clinical manifestations, types of drugs involved, and the therapeutic interventions administered. The severity of poisonings was evaluated using the Poisoning Severity Score, and toxicological analysis was performed using gas chromatography–mass spectrometry. **Results:** The results indicated that poisonings were most prevalent in adolescent girls (72%), with 78% of cases resulting from intentional poisoning, while unintentional poisoning was more common in children. Benzodiazepines, antipsychotics, and analgesics were the primary drugs causing these poisoning incidents. The majority of patients (78%) experienced mild clinical symptoms, whereas 9% of pediatric patients suffered from severe poisoning, related to complications such as aspiration pneumonia and acute renal failure. Addressing pediatric drug poisoning in Vojvodina requires an increased focus on preventive strategies, including parental education and appropriate psychosocial support for the youth. **Conclusions:** Through collaborative efforts among healthcare providers, educators, and policymakers, prevention, treatment, and support mechanisms can be enhanced to combat this pressing public health challenge.

## 1. Introduction

Drug poisoning in pediatric populations is a major global public health issue and one of the leading causes of admissions to pediatric emergency departments [1,2]. Although drug poisoning may result in mild clinical symptoms, in some instances it can lead to severe complications and fatal outcomes [3]. According to the World Health Organization (WHO), acute poisoning causes approximately 45,000 deaths annually in children and young people under the age of 19 [2]. Understanding the etiology of poisoning and implementing preventive measures can help reduce the incidence of this condition and increase public awareness [1]. According to the National Poison Control Centre of the Military Medical Academy (MMA), 142 emergency calls due to possible poisoning of children and adolescents by medicines were registered in Serbia during 2023 [4]. The causes and types of poisoning vary considerably across countries and regions. Therefore, each country or region must assess its specific epidemiological landscape with regards to poisoning incidents, including their respective causes and types, so that preventive measures and interventions can be implemented, aiming at the reduction of the poisoning incidence [2].

The annual report from Serbia’s National Poison Center does not provide specific data on pediatric poisonings [4]. Additionally, there is only a limited number of studies in Serbia about drug poisoning in the pediatric population [5,6,7]. Recent studies in Europe confirm that pediatric drug poisonings represent a significant clinical and public health concern, with exposure patterns varying according to age and intent. Accidental ingestions are predominant among younger children, most commonly involving pharmaceuticals such as antibiotics, paracetamol, and benzodiazepines. In contrast, intentional self-poisoning is more frequently observed in adolescents and is often associated with psychiatric disorders or self-harm attempts [8,9,10]. Clinical outcomes range from mild symptoms to severe complications requiring intensive care, including respiratory depression, hepatic failure, and neurological impairment. Several studies have identified benzodiazepines, antidepressants, and over-the-counter analgesics as the most frequently implicated substances, with benzodiazepines particularly associated with central nervous system depression and potentially life-threatening outcomes in overdose cases [8,9,11]. Furthermore, recent data indicates an increasing trend in adolescent self-poisoning, exacerbated by psychosocial factors and limited access to mental health care institutions, highlighting the urgent need for targeted preventive interventions within vulnerable pediatric populations [12].

The aim of this study is to investigate the prevalence of drug poisoning among hospitalized children and adolescents in Vojvodina over the period from 2018 to 2023. More specifically, this study aims to investigate the demographic and clinical features of acute poisoning cases, identify the most commonly involved drugs, evaluate the poisonings’ severity and outcomes, and examine the trends over the mentioned period, including the potential impact of the COVID-19 pandemic. Taking into consideration that the diagnosis of poisoning in the patient’s medical history is often unreliable, it has been shown that the blood toxicological analysis is a valuable tool in resolving different causes of poisoning [13,14,15]. Thus, this study also aims to explore the role and reliability of toxicological analyses in investigating pediatric poisonings, along with the information obtained from the patients.

## 2. Materials and Methods

### 2.1. Study Population

A retrospective, observational study was conducted in accordance with the STROBE (Strengthening the Reporting of Observational Studies in Epidemiology) guidelines at the Institute for Child and Youth Health Care of Vojvodina and the Centre for Forensic Medicine, Toxicology, and Molecular Genetics in Novi Sad, Serbia, covering the period from 2018 to 2023. This study was approved by the Ethics Committees of these institutions and conducted in line with the principles of the Declaration of Helsinki. We used the Health information system to identify acute drug poisoning cases using ICD-10 classification codes T36.0–T50.0. Toxicological analyses of biological samples from pediatric-age patients under the age of 18 were performed in the Centre for Forensic Medicine, Toxicology, and Molecular Genetics based on the suspicion of acute poisoning. This study included acutely poisoned pediatric-age patients, who were hospitalized at the Institute for Child and Youth Health Care of Vojvodina, after a thorough review of their medical records, including toxicological analysis data supporting the poisoning. The inclusion of cases in this study required analytical confirmation of poisoning through toxicological analysis. Cases with incomplete medical documentation and/or missing data on study variables (described below) were excluded from this study.

### 2.2. Data and Study Variables

To determine the characteristics and estimated degree of poisoning in the pediatric population, the following information was collected from each patient’s medical documentation by two independent, experienced clinical toxicology researchers. Inconsistencies were resolved through consensus discussions, and all data entered was adhering to the standard operating procedures implemented at the hospital. Medical records, including toxicological analyses and clinical documentation, were carefully reviewed, coded and imported in a Microsoft Excel (Seattle, WA, USA) spreadsheet.

The study variables within our dataset were defined as follows: demographic characteristics (age, gender, and area of residence), number and type of exposure to drugs, manner of poisoning (intentional or unintentional), cause of poisoning, repeated poisoning, drug concentration in the whole blood, clinical manifestations of poisoning (signs and symptoms of poisoning with objective clinical effects such as adynamia, drowsiness, unconsciousness, coma, somnolence, convulsions or respiratory arrest), Poisoning Severity Score (PSS), initial vital signs (pulse, respiration, blood pressure), therapeutic procedures used, complications during the treatment (aspiration pneumonia, renal insufficiency), and length of hospitalization (days).

The cases were categorized as children (<10 years old), early (10–13 years old), middle (14–16 years old), and late adolescents (17 years old) [16]. Vital signs were recorded as initial values during admission. Increases or decreases in blood pressure and heart rate are defined as age-related in the pediatric population [17]. Gas chromatography with a mass spectrometry detector (GC-MS) was used to determine drug concentrations in biological samples of poisoned patients, according to a previously described procedure [18]. Drug concentrations were determined using calibration curves constructed from external standards analyzed under identical experimental conditions (Supplementary Materials). We evaluated the alignment between the results of the toxicological analyses and the information provided from the medical history about the patients’ exposure to drugs.

The Poisoning Severity Score (PSS) assessed the poisoning based on clinical signs and independently of the dose, concentration, type, and number of poisoning agents [19]. The Poisoning Severity Score consisted of four levels: 0—no symptoms and features of poisoning; 1—minor poisoning (minor symptoms and features of poisoning); 2—moderate poisoning (marked or extended symptoms and features of poisoning); 3—severe poisoning (severe and life-threatening symptoms and features of poisoning); 4—fatal outcome.

### 2.3. Statistical Analysis

The obtained results were processed using Microsoft Office Excel (v2019) and SPSS (version 26.0 for Windows, SPSS, Chicago, IL, USA). The results were analyzed using descriptive statistics. Continuous variables are presented as median or mean values, while categorical variables are expressed as percentages of category frequencies. The following hypotheses were tested to assess the differences and relationship among various factors related to pediatric drug poisoning. The Chi-square test was used to test whether there was a significant difference in the intent of poisoning between children and adolescents. Also, the Chi-square was used to test whether there were significant differences in poisoning intent based on patients’ area of residence and gender. A multinomial goodness-of-fit test was used to determine whether the distribution of poisoning cases over the six years (2018–2023) significantly deviated from the expected proportions of total hospitalizations for each year. Spearman’s rank correlation was used to test the correlation between the length of hospital stay and the Poisoning Severity Score (PSS), and between the concentration of exposed drugs and the Poisoning Severity Score (PSS). All the analyses were evaluated at the level of a statistical significance of 0.05.

## 3. Results

**Figure 1:**
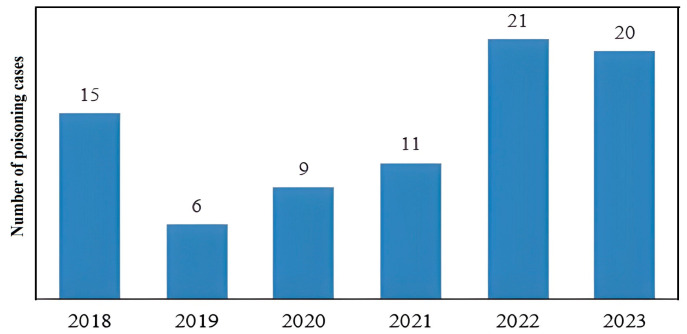
Poisoning cases documented by the Institute for Child and Youth Health Care of Vojvodina during a six year period (2018–2023).

Out of 253 cases with suspicion of acute drug poisoning in pediatric patients from 2018 to 2023, the presence of exposure drugs was confirmed by toxicological analysis in 82 cases (32.66%). The data for each year over the analyzed period indicates a rise in poisoning cases among the pediatric population starting from 2019, which continued following the onset of the COVID-19 pandemic (*p* = 0.02) (Figure 1).

Cases were categorized as children (age 0–9) and adolescents (age 10–17) to describe the main socio-demographic differences between patients and circumstances of poisoning in the pediatric population (Table 1). The majority of cases (78%) were related to intentional poisoning, while unintentional poisonings accounted for 22% of the cases. The results indicate a higher prevalence of poisoned patients in the pediatric population among females (72%) compared to males (*p* < 0.001). Moreover, most of the pediatric patients affected by intentional poisoning (70%) originated from urban areas rather than rural regions (*p* < 0.001). The circumstances leading to poisoning were associated with suicide attempts (34%), family conflicts (31%), peer violence (13%), and unintentional reasons (22%). In the studied population, 82% of cases involved a first-time poisoning case, while 18% included patients with a previous poisoning attempt, all of which were from the intentional poisoning group.

**Figure 2:**
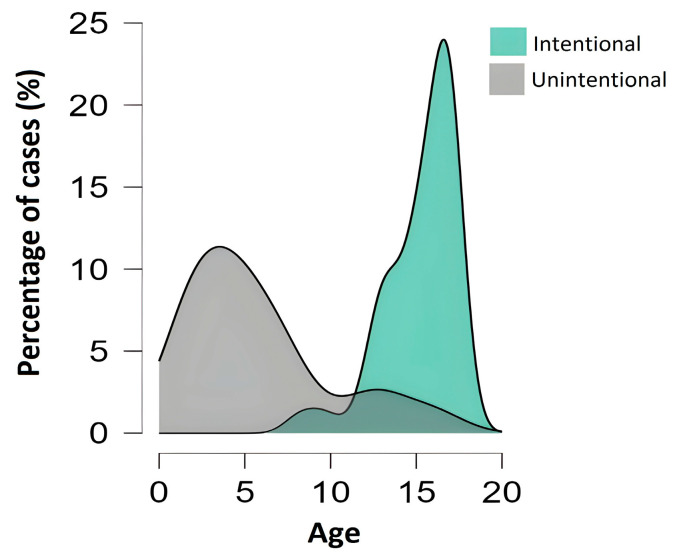
Age distribution (%) of intentional and unintentional poisoning cases.

Most poisonings in children with a median age of 4.5 (IQR = 4.75) were unintentional, whereas in adolescents with a median age of 16 (IQR = 3.00), most poisonings were intentional (Figure 2). The age distribution of poisoning cases indicated that pediatric patients aged 14–17 accounted for 62% of the cases. Among adolescents, the largest number of intentional poisonings was observed in middle adolescence (ages 13–16) (43%), followed by late adolescence (age 17) (36%) and early adolescence (ages 10–12) (21%).

The main clinical features and manifestations of poisoning cases are summarized in Table 2. Approximately 78% of pediatric patients with poisoning had mild symptoms (PSS = 1) that required minimal treatment or observation. Moderate poisoning (PSS = 2) was observed in 13% of the cases, while a severe clinical picture (PSS = 3), which required prolonged treatment, was observed in 9% of the cases. The data provided in Table 2 also presents the number of ingested substances and the alignment between patient-reported exposure to drugs and toxicological analysis results. This comparison highlights the comparability of patient-provided information with toxicological results.

Most patients received symptomatic treatment with iv solutions and non-specific detoxification therapy. Gastric lavage was performed in 40% of the cases to prevent further absorption of the poison, while activated carbon was administered in 44% of the cases. Specific antidotes were used in a few instances: flumazenil was administered in four cases to treat benzodiazepine poisoning, which resulted in a positive outcome. More aggressive interventions, such as intubation and mechanical ventilation, were required in 7% of the cases. Aspiration pneumonia was noted in 16% of the cases and resulted in prolonged hospitalization.

**Figure 3:**
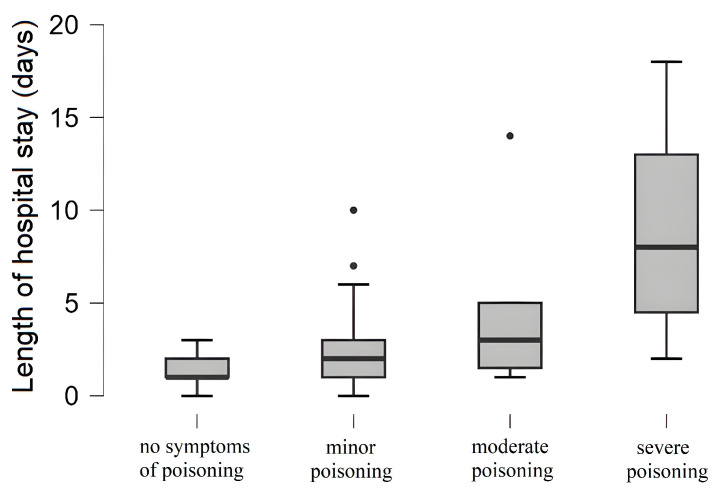
Box and whiskers plot of the length of hospital stay based on different Poisoning Severity Score (PSS) categories: no symptoms (0), minor (1), moderate (2), and severe poisoning (3).

A significant positive correlation was found between the duration of hospitalization and the severity of poisoning (rho = 0.652; *p* < 0.001) (Figure 3). The mean length of hospitalization for patients with a PSS of 3 was 9 days.

**Figure 4:**
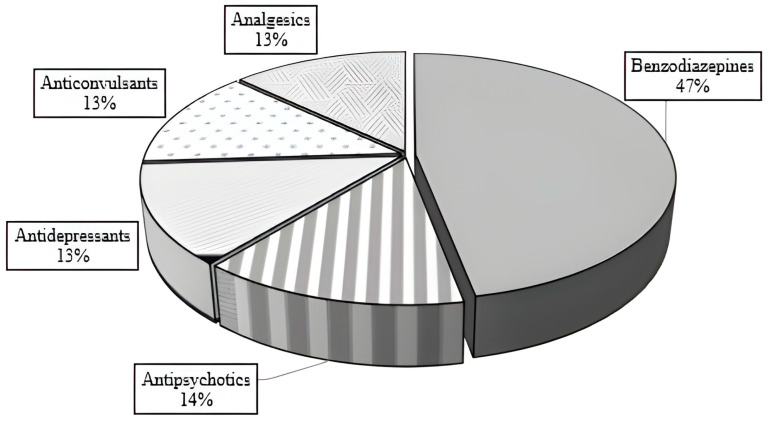
Pharmacological agents involved in poisoning.

The data showed that poisoning in pediatric patients most frequently involved the following drug groups: benzodiazepines (47%), followed by antipsychotics, antidepressants, anticonvulsants, and analgesics (Figure 4). Intentional poisoning with benzodiazepines was most frequently caused by bromazepam (20 cases), diazepam (15), lorazepam (5), clonazepam (3), and alprazolam (1). Poisoning with antipsychotics was recorded with olanzapine (6), clozapine (3), quetiapine (2), and haloperidol (2). Most of the poisoning cases related to anticonvulsants were caused by carbamazepine (8), followed by lamotrigine (2) and valproic acid (2). Among antidepressant poisonings in pediatric patients, sertraline poisoning was the most common (6), followed by citalopram (2) and fluoxetine (2). Poisoning cases involving analgesics in pediatric patients were most frequently recorded with ibuprofen (9), followed by paracetamol (3) and diclofenac (2).

Table 3 presents the results of the toxicological analysis, including the median drug concentrations in the patient’s blood.

Table 4 provides an overview of the clinical characteristics of pediatric patients with poisoning, highlighting the differences among groups of pharmacological agents. In cases of benzodiazepine poisoning in pediatric patients, a mild clinical presentation with a stable overall condition was observed in 91% of patients upon admission. In contrast, more severe cases, including coma, were recorded in two patients (5%). Benzodiazepines cause central nervous system (CNS) depression, so the most common clinical effects were drowsiness (44%), adynamia (28%), unintelligible speech, ataxia, and miosis. In the reported cases of pediatric benzodiazepine poisoning, hypotension (44%) was commonly observed, accompanied by compensatory tachycardia, which required treatment for the stabilization of vital functions.

Poisoning with analgesics included with ibuprofen and paracetamol poisoning cases. The most common symptoms of analgesic poisoning included nausea and vomiting (67%), as well as abdominal pain. Drowsiness was observed in cases of combined poisoning with CNS depressants. Among clinical features, hypotension occurred in 42% of cases, often accompanied by compensatory tachycardia. Acute renal insufficiency was developed in two instances of ibuprofen poisoning, one of which progressed to coma.

Poisoning cases with antidepressants predominantly involved selective serotonin reuptake inhibitors (SSRIs), with common symptoms including nausea, vomiting, loss of consciousness, anxiety, and tremors. Tachycardia was observed in 83% of cases; however, no changes were noted on the electrocardiogram.

Cases of poisoning involving antipsychotics were most commonly associated with atypical antipsychotics. The most prevalent clinical effect was loss of consciousness (46%), which often occurred in conjunction with the use of CNS depressants. Other observed effects included tachycardia (77%), tachypnea, nausea, vomiting, drowsiness, and disorientation.

The most severe cases of poisoning in pediatric patients from this study were caused by anticonvulsant drugs, particularly carbamazepine. Clinical manifestations included coma (50%), nausea, and vomiting, leading to aspiration pneumonia in 58% of cases. Additional signs included narrow, non-reactive pupils in response to light. A strong positive correlation was found between carbamazepine concentration and the severity of poisoning (rho = 0.730; *p* = 0.04).

## 4. Discussion

The study results indicate a statistically significant increase in pediatric poisoning cases starting from 2019, persisting through the pandemic years, as confirmed by a multinomial test comparing the observed rise to expected annual hospitalization proportions. While the number of cases recorded in 2018 introduces variability, the increase starting from 2019 suggests that factors predating the pandemic, such as changes in medication availability or societal stressors, may have contributed to this trend, with the pandemic likely acting as an exacerbating factor through the COVID-19-related social changes. These findings are consistent with prior research suggesting that prolonged stay-at-home orders and school absences during COVID-19 episodes may have elevated the risk of pediatric poisoning [20]. Furthermore, COVID-19 has been linked to the worsening of mental health issues [21] and an increase in the use of medications such as benzodiazepines in adults, making these drugs readily available at home [22]. The observed increase should be interpreted cautiously, considering broader social and temporal factors.

Our findings substantiate the importance of toxicological analysis as a supplement to the information provided by patients or their companions, particularly in pediatric poisoning cases, where the available information is often incomplete or unreliable. Previous research has primarily been based on the patient’s medical history and frequently not supported by toxicological evidence [23]. The results of initial urine screening should be interpreted with caution until being confirmed by GC-MS toxicological analysis, as there is a risk of false positive results [24]. The misalignment between patient-reported information and toxicological results in pediatric poisoning cases highlights the crucial role of toxicological analyses in clinical practice. In 59% of cases involving children, caregiver-provided information did not align with toxicological findings, likely due to the accidental nature of most poisonings and uncertainty about the ingested substances. In contrast, alignment among adolescents was higher, with discrepancies observed in 28% of cases compared to self-reported data. The observed drug concentrations in our study were lower than the toxic levels reported in the literature for adults [25].

The rate of drug poisoning in the pediatric population in Vojvodina is 0.59 cases of poisoning per 10,000 children per year. However, to the best of our knowledge, no similar epidemiological studies have been conducted in Serbia or neighboring countries, which limits the possibility of direct regional comparisons. A much higher incidence of poisoning was found in Washington, USA, with up to 4.5 poisonings per 10,000 children per year [26]. The results indicate a higher prevalence of poisoned patients in the pediatric population among females compared to males, which is comparable with the findings from previous studies [1,2,26,27]. A study by Dündar et al. (2021) reported that 70% of all poisonings among adolescents were intentional self-poisonings [2]. Gauvin et al. (2001) have also shown that drug poisoning is one of the most common methods used for suicide attempts among adolescents [26]. The results of our study align well with the previous research, as 34% of adolescent poisonings were confirmed as intentional attempts of self-harm. Poisoning continues to represent one of the leading causes of mortality among adolescents, as reported in recent literature, which indicates the importance of preventive efforts and targeted interventions [28,29]. Similar to the previous studies, our findings confirm that the age distribution in cases of poisoning among the pediatric population follows a bimodal pattern [1,30,31] and that there is a positive correlation between the duration of hospitalization and the severity of poisoning [26].

Most of the pediatric patients affected by intentional poisoning were from urban areas, whereas cases in rural regions were predominantly unintentional. According to previous research, the predominance of urban cases likely reflects better access to healthcare, but also higher exposure to mental health stressors and medications in adolescents [2,32]. In contrast, rural regions face challenges such as unsafe medication storage and limited caregiver education [31], emphasizing the need for targeted prevention strategies in both settings.

This research’s data showed that poisoning in pediatric patients most frequently was caused by benzodiazepines. While the comprehensive epidemiological data on poisoning in the pediatric population is lacking in our country, we observed that nearly half of the cases were caused by benzodiazepines (47%), while antipsychotics (14%), antidepressants (13%), anticonvulsants (13%), and analgesics (13%) were almost equally represented. Previous retrospective studies indicated that poisoning in the pediatric population most commonly involves analgesics and CNS agents [1,2,26,30,33,34]. Benzodiazepines are widely used and readily accessible, making them a common agent in cases of pediatric poisoning. Flumazenil, a specific antidote, was administered in three cases, all of which resulted in a positive response. Particular caution is required when administering flumazenil to children with a history of epilepsy attacks or cardiac conditions [30]. An increasing trend in benzodiazepine use has been observed in Serbia, with bromazepam, diazepam, lorazepam, and alprazolam being the most commonly used [35]. Samardžić et al. (2018) also showed irrational prescription of drugs and benzodiazepine abuse in our population [35]. Among antidepressants, poisoning cases have predominantly involved SSRIs. While SSRIs are known for their high therapeutic index and low toxicity in children [36], they can still lead to serious side effects, including serotonin syndrome [37]. A study by Klein-Schwartz et al. (2012) highlighted citalopram being associated with an increased risk of cardiotoxicity in pediatric poisoning cases [38]. Additionally, a recent study indicated that SSRIs are the most frequently reported medications in cases of unintentional ingestion, with 68% of these cases showing no symptoms [39]. In the pediatric population, poisoning with antipsychotics is typically mild to moderate, with neurological manifestations such as CNS depression and extrapyramidal symptoms [40,41,42].

With regards to analgesics, in one case, a young girl developed acute renal insufficiency after ingesting 11.5 g of ibuprofen, as reported in the medical history from our study. Another case involved a girl with combined analgesic poisoning (naproxen and ibuprofen) who developed acute renal insufficiency, pancreatitis, and cholelithiasis. Overall, ibuprofen poisoning resulted in acute renal insufficiency in two cases, with one progressing to coma. These findings, consistent with similar studies, demonstrate that ibuprofen overdose can lead to severe complications. Consequently, careful monitoring of renal function is essential in patients with overdoses [43,44,45]. The higher prevalence of ibuprofen-related poisonings in our study, compared to paracetamol, which is often reported as the leading cause of poisoning in other studies, may reflect regional prescription patterns and the high availability and use of ibuprofen for pain management in our study population. The most severe cases of child poisoning recorded in this study involved anticonvulsant drugs, particularly carbamazepine, which resulted in significant clinical complications. A strong positive correlation was identified between carbamazepine concentration and the severity of poisoning, consistent with previous studies [30].

In our study, gastric lavage was performed in 40% of the cases and aligned well with the current clinical guidelines, with gastric lavage being conducted within 1–2 h after the ingestion of the toxic substances, the time frame during which it is considered the most effective [45]. This timeframe reflects the typical admission of patients to the hospital after exposure.

In accordance with the presented results and the vulnerability of the pediatric patient population, it is essential to develop preventive strategies to reduce instances of poisoning. We recommend the implementation of unit dose packaging [46], where medications or substances are divided into individual doses, each sealed separately. In addition, child-resistant packaging [47] is critical in preventing children from accessing medication. Furthermore, medications should be stored in locked cabinets at locations inaccessible to children, with the cap securely resealed after each use to ensure safety further [48]. Further awareness about this issue must be raised through parent education [49]. It is essential to establish and train teams focused on mental health protection, as well as developmental counseling centers that can provide psychological and psychiatric support to adolescents. These teams should focus on both prevention and follow-up care after poisoning incidents, as repeated poisonings account for nearly one-fifth of the cases recorded in this study. This support should be integrated within schools and healthcare facilities to ensure accessibility and effectiveness [50].

All patients were discharged from the Pediatric Clinic of the Institute for Child and Youth Health Care of Vojvodina after hospitalization. Consequently, advisory discussions were held with parents to promote preventive measures against unintentional poisoning from medications and household substances. The collaboration between the Toxicology Laboratories, the Clinical Center of Vojvodina, and the Institute for Child and Youth Health Care of Vojvodina represents a model of good medical practice and effective cooperation in managing pediatric poisoning cases.

This study has inherent limitations typical for retrospective designs, including reliance on medical records, which may introduce imperfection, particularly from excluding cases without toxicological confirmation or complete documentation. Additionally, factors such as variability in drug metabolism, timing of sample collection, and hydration status may have influenced the observed discrepancies between clinical presentations and toxicological results. This study’s focus on a single region and a relatively modest sample size could limit the transferability of findings to the wider population. Furthermore, as all included cases were observed in a healthcare facility, it remains unknown what percentage of poisoning cases actually make it to a health care facility for management. However, these limitations are balanced by this study’s notable strengths. It employs robust methodologies, including standardized tools such as the Poisoning Severity Score and GC-MS analyses, ensuring reliability and reproducibility. This study addresses an important gap in the literature by providing detailed toxicological and clinical data specific to pediatric poisonings in Serbia, offering actionable insights for public health policies and preventive interventions. These contributions lay a solid groundwork for prospective studies exploring the long-term impacts and prevention of pediatric poisonings.

## 5. Conclusions

This study provides a comprehensive overview of the prevalence of poisoning among hospitalized children and adolescents in Vojvodina over the period from 2018 to 2023, providing detailed insights into the demographic and clinical features of affected patients. It identifies benzodiazepines as the most frequently involved drugs, followed by antipsychotics and analgesics, and highlights the predominance of intentional poisonings among adolescents, with unintentional cases more common in children. The results also reveal that poisoning severity is mostly mild, but the severe cases are linked to complications like aspiration pneumonia and acute renal failure. The results also indicate an increasing trend in poisoning cases beginning in 2019, with a notable rise during the COVID-19 pandemic, likely reflecting broader societal and psychological challenges. Furthermore, the results validate the importance of toxicological analysis in complementing patient-reported information, particularly in pediatric cases where such information is often incomplete or inconsistent.

## Figures and Tables

**Table 1:** Demographic characteristics of the study population: differences between children and adolescents in poisoning cases.

	All Cases	Children (<10 Years)	Adolescents (10–17 Years)
n (%)	82	17 (21%)	65 (79%)
Gender			
Male	23 (28%)	9 (53%)	15 (23%)
Female	59 (72%)	8 (47%)	50 (77%)
Age (year; IQR)			
Median		4.5 (4.75)	16 (3)
Manner of poisoning			
Intentional	64 (78%)	2 (2%)	62 (95%)
Unintentional	18 (22%)	15 (88%)	3 (5%)
Area			
City	53 (64%)	8 (47%)	45 (69%)
Rural	29 (36%)	9 (53%)	20 (31%)
Repeated poisonings			
No	67 (82%)	17 (100%)	49 (76%)
Yes	15 (18%)	NR	15 (24%)
Cause of poisoning			
Suicide attempt	29 (34%)	NR	29 (45%)
Conflict in the family	26 (31%)	1 (6%)	25 (38%)
Peer violence	9 (13%)	1 (6%)	8 (12%)
Unintentional	18 (22%)	15 (88%)	3 (5%)

n (%)—number of patients (percentage); IQR—interquartile range; NR—Not Reported.

**Table 2:** Clinical features of the study population, number of ingested substances, and alignment between patient-reported exposure to drugs and toxicological results.

	All Cases	Children (<10 Years)	Adolescents (10–17 Years)
Poisoning Severity Score			
1	64 (78%)	13 (76%)	51 (78%)
2	11 (13%)	3 (18%)	8 (12%)
3	7 (9%)	1 (6%)	6 (10%)
Number of ingested substances			
1	56 (68%)	13 (76%)	42 (66%)
2	16 (19%)	3 (18%)	13 (20%)
3	10 (13%)	1 (6%)	9 (14%)
Is there an alignment between patient-reported drug exposure and toxicological analysis results?			
Yes	53 (59%)	7 (41%)	46 (72%)
No	29 (41%)	10 (59%)	18 (28%)

**Table 3:** Drug concentrations in patient’s blood.

	n	C_median_ (µg/mL)	Minimum	Maximum	LOD (µg/mL)	LOQ (µg/mL)
Bromazepam	20	0.095	0.010	0.689	0.001	0.010
Diazepam	15	0.202	0.061	0.891	0.022	0.060
Ibuprofen	9	1.784	0.262	8.277	0.172	0.250
Carbamazepine	8	9.789	2.432	33.44	0.458	1.520
Sertraline	6	0.186	0.023	1.855	0.001	0.012
Lorazepam	5	0.044	0.065	0.075	0.005	0.060
Paracetamol	3	1.478	0.120	1.905	0.010	0.100
Clonazepam	3	0.037	0.034	0.040	0.001	0.010
Haloperidol	2	0.042	0.037	0.050	0.001	0.010
Citalopram	2	0.099	0.012	0.186	0.001	0.011
Lamotrigine	2	10.28	10.80	12.74	0.263	1.200
Fluoxetine	1	0.278	0.278	0.278	0.005	0.017

n—number of cases; C_median_—concentration of drugs in patient’s blood expressed as median (µg/mL); Minimum and Maximum—minimal and maximal determined concentration expressed as µg/mL in these study cases; LOD—limit of detection drugs using GC-MS method expressed as µg/mL; LOQ—limit of quantification drugs using GC-MS method expressed as µg/mL.

**Table 4:** Clinical features of the study population and differences among groups of pharmacological agents.

	Benzodiazepines	Antidepressants	Antipsychotics	Anticonvulsants	Analgesics
n	43	12	13	12	12
Poisoning Severity Score					
1	39 (91%)	9 (76%)	9 (69%)	6 (50%)	10 (84%)
2	3 (7%)	2 (16%)	3 (23%)	2 (17%)	1 (8%)
3	1 (2%)	1 (8%)	1 (8%)	4 (33%)	1 (8%)
Clinical manifestation					
Adynamia	12 (28%)	3 (25%)	2 (15%)	2 (17%)	1 (8%)
Sleepiness	19 (44%)	1 (8%)	3 (23%)	2 (17%)	3 (25%)
Unconsciousness	5 (12%)	4 (33%)	6 (46%)	6 (50%)	1 (8%)
Nausea	5 (12%)	4 (33%)	4 (32%)	2 (17%)	8 (67%)
Consciousness					
Coma	2 (5%)	1 (8%)	1 (8%)	6 (50%)	2 (17%)
Somnolence	7 (16%)	1 (8%)	3 (23%)	2 (17%)	1 (8%)
Conscious	34 (79%)	9 (75%)	9 (69%)	4 (33%)	9 (75%)
Vital parameters					
Tachycardia	23 (53%)	10 (83%)	10 (77%)	8 (67%)	7 (58%)
Bradycardia	4 (9%)	NR	1 (8%)	NR	2 (17%)
Hypertension	NR	1 (8%)	1 (8%)	NR	1 (8%)
Hypotension	19 (44%)	3 (25%)	4 (31%)	4 (33%)	5 (42%)
Tachypnea	10 (23%)	2 (17%)	4 (31%)	6 (50%)	4 (33%)
Bradypnea	3 (7%)	NR	1 (8%)	2 (17%)	1 (8%)

NR—Not Reported.

## Data Availability

The data are available from the corresponding author upon request.

## Supplementary Materials

The following supporting information can be downloaded at: https://www.mdpi.com/article/10.3390/jcm14175967/s1, Figures S1–S12: Calibration curve of drug standards (Rt = min, m/z).

## Abbreviations

The following abbreviations are used in this manuscript:

WHO	World Health Organization
MMA	Military Medical Academy
STROBE	Strengthening the Reporting of Observational Studies in Epidemiology
ICD-10	International Classification of Diseases, 10th Revision
GC-MS	Gas Chromatography–Mass Spectrometry
PSS	Poisoning Severity Score
SSRIs	Selective Serotonin Reuptake Inhibitors
CNS	Central Nervous System
SPSS	Statistical Package for the Social Sciences
