# Supplementary material for: Pediatric Drug Poisoning in Vojvodina, Serbia: A Retrospective Observational Clinical and Toxicological Assessment
Source: J Clin Med. 2025 Aug 23;14(17):5967. doi: 10.3390/jcm14175967 (PMC12429275; doi:10.3390/jcm14175967)
Supplement: Supplementary file 1 [file jcm-14-05967-s001.zip › jcm-3754571-supplementary.pdf]

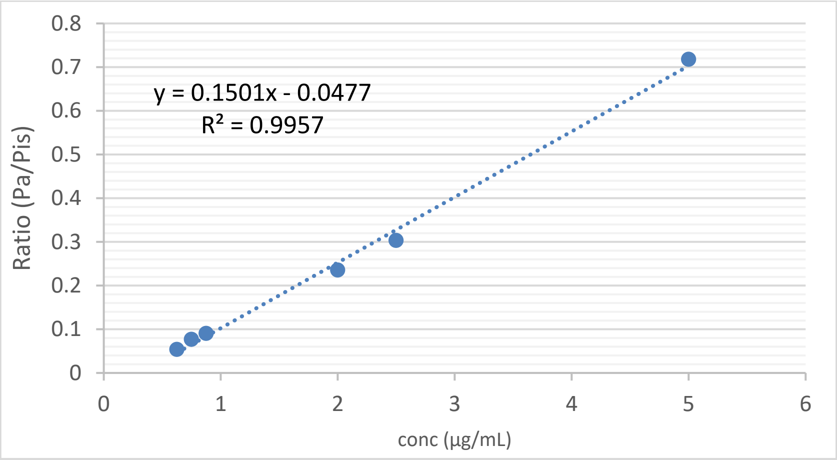

Figure S1. Calibration curve of bromazepam standard (Rt=13,2 min, m/z=315)

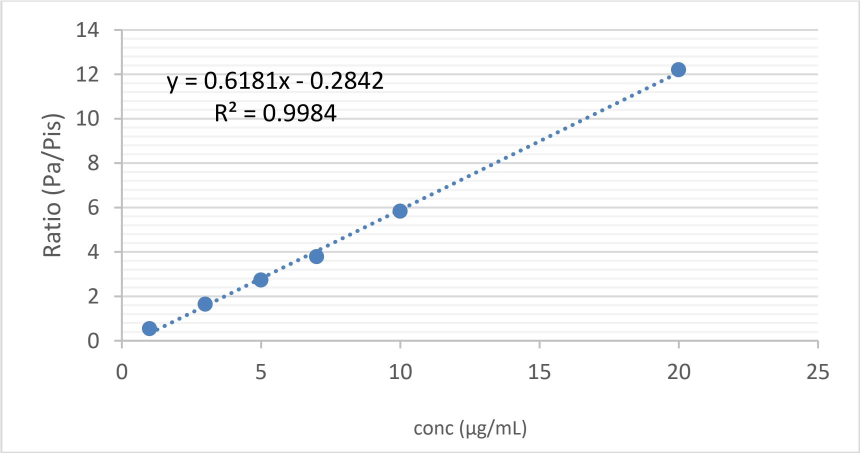

Figure S2. Calibration curve of diazepam standard (Rt=12,21 min, m/z=283)

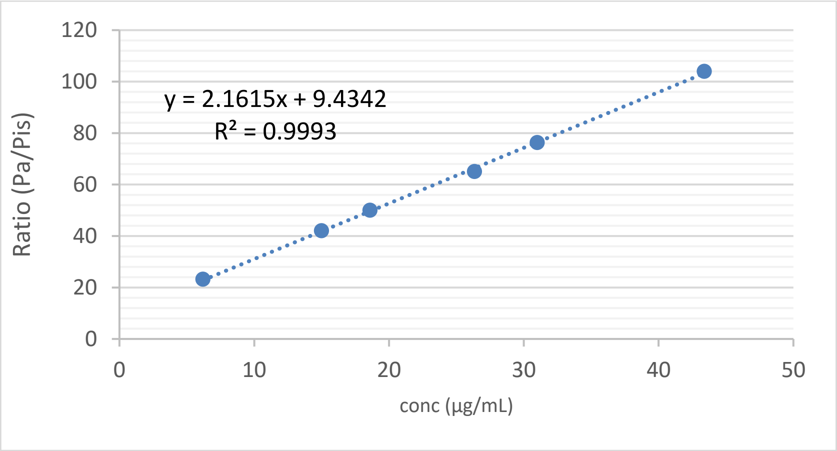

Figure S3. Calibration curve of ibuprofen standard (Rt=7,01 min, m/z=161)

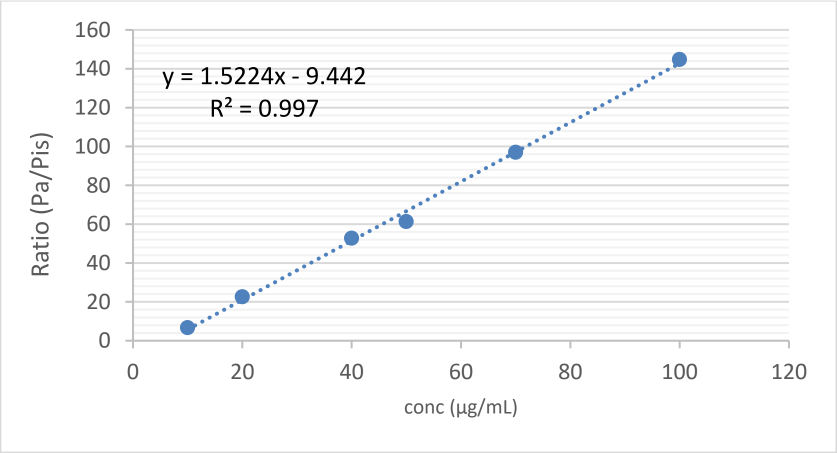

Figure S4. Calibration curve of carbamazepine standard (Rt=10,5 min, m/z=193)

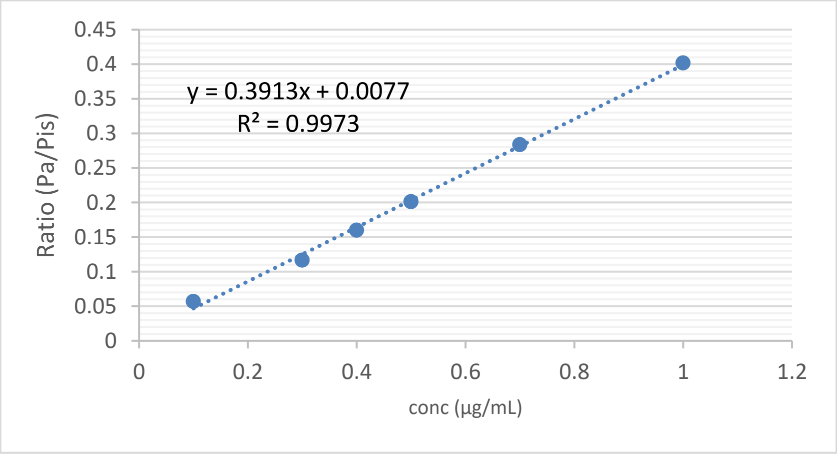

Figure S5. Calibration curve of sertraline standard (Rt=11,80 min, m/z=274)

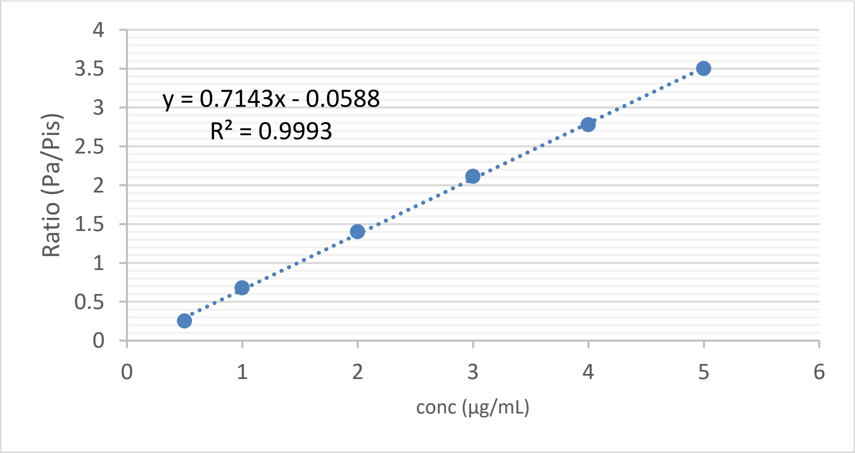

Figure S6. Calibration curve of lorazepam standard (Rt=12,00 min, m/z=302)

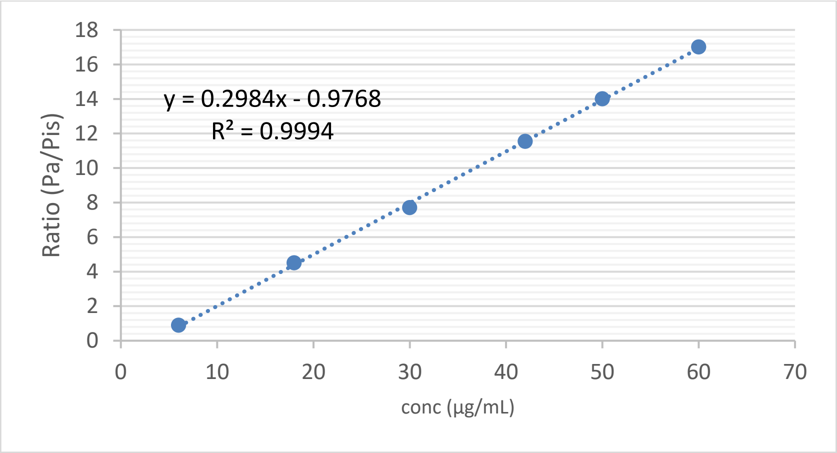

Figure S7. Calibration curve of paracetamol standard (Rt=6,80 min, m/z=151)

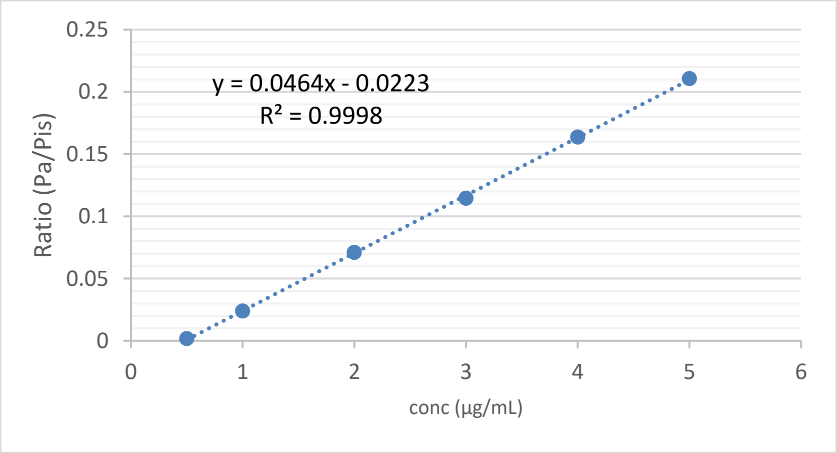

Figure S8. Calibration curve of clonazepam standard (Rt=13,90 min, m/z=314)

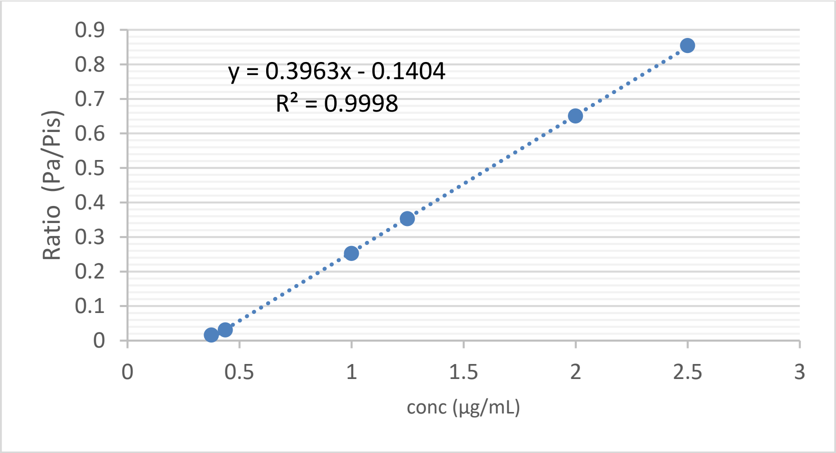

Figure S9. Calibration curve of haloperidol standard (Rt=15,56 min, m/z=224)

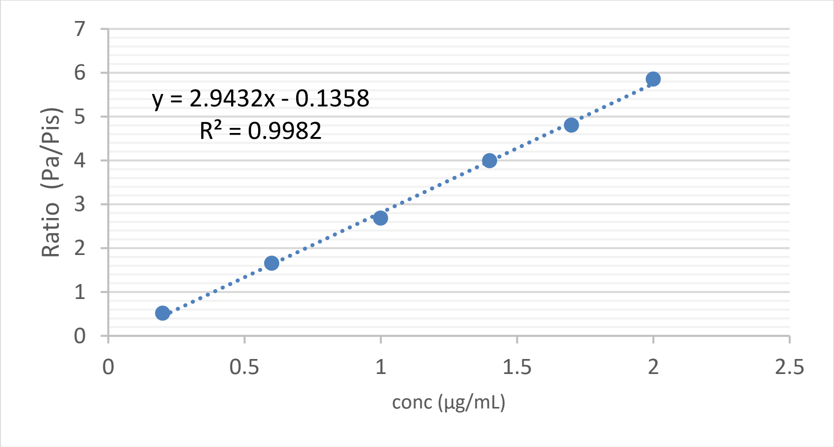

Figure S10. Calibration curve of citalopram standard (Rt=11,96 min, m/z=58)

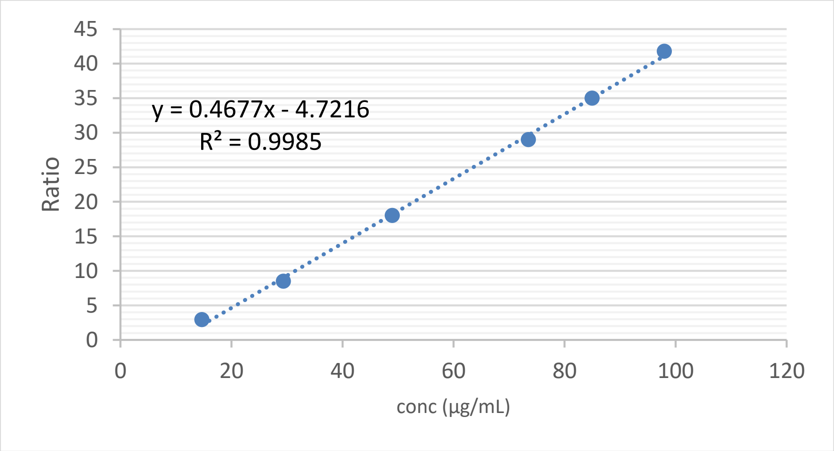

Figure S11. Calibration curve of lamotrigine standard (Rt=11,50 min, m/z=185)

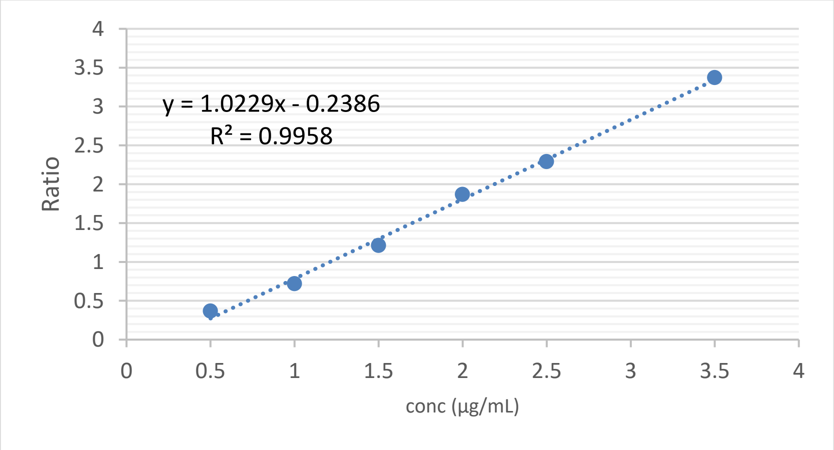

Figure S12. Calibration curve of fluoxetine standard (Rt=8,42 min, m/z=44)
